# Effect of a LoBAG_30_ diet on protein metabolism in men with type 2 diabetes. A Randomized Controlled Trial

**DOI:** 10.1186/1743-7075-9-43

**Published:** 2012-05-20

**Authors:** Frank Q Nuttall, Mary C Gannon

**Affiliations:** 1 Section of Endocrinology, Metabolism & Nutrition, Minneapolis VA Health Care System (FQN, MCG), University of Minnesota, Minneapolis/St. Paul, Minnesota, USA; 2 Departments of Medicine (FQN, MCG) and Food Science & Nutrition (MCG), University of Minnesota, Minneapolis/St. Paul, Minnesota, USA; 3 Chief, Endocrinology, Metabolism & Nutrition Section, Professor of Medicine, Minneapolis VA Medical Center, One Veterans Drive 111G, Minneapolis, MN, 55417, USA

**Keywords:** LoBAG diet, Protein balance, Nitrogen balance, Fat-free mass, Body composition, Amino acids, Cortisol, Glucagon, Sarcopenia

## Abstract

**Background:**

We previously reported that a weight-maintenance diet with a carbohydrate:protein:fat ratio of 30:30:40%, ingested for 5 weeks, improved blood glucose control in subjects with untreated type 2 diabetes. In this study we also determined that insulin and insulin-like growth factor-I (IGF-I) were increased. In this report we provide further information. Specifically, 24-hour total and individual amino acids, glucagon and cortisol data are provided. In addition, we determined whether these multiple effectors resulted in a positive nitrogen balance and an increase in fat-free mass. Insulin and IGF-I should stimulate protein accumulation. An increase in amino acids, particularly branched chain amino acids, should facilitate this, whereas glucagon and cortisol could have adverse effects in this regard.

**Methods:**

Eight men with untreated type 2 diabetes were studied. A randomized crossover design was used. Data were obtained before and after 5 weeks on a control diet (55% carbohydrate:15% protein:30% fat) and on a 30% carbohydrate:30% protein:40% fat diet. Nitrogen balance and body composition were determined at the beginning and end of each dietary intervention.

**Results:**

As expected, the mean 24-hour total amino acid area response was higher after ingesting the 30:30:40 diet. However, the increase was only statistically significant for the branched chain amino acids, and phenylalanine and tyrosine. The 24-hour cortisol profile was unchanged. Glucagon was increased. Nitrogen balance was positive. Body weight was stable. Body composition and computed tomography data indicate no change in the fat-free mass.

**Conclusion:**

This high protein, low carbohydrate diet induced a metabolic milieu which strongly favors a positive protein balance, and a positive balance was present. However, an increase in lean (protein) mass was not documented. Whether such a diet in people with type 2 diabetes is useful in preventing or delaying the loss of total lean body mass and/or sarcopenia associated with aging remains to be determined.

## Background

We have designed diets referred to as Low Biologically Available Glucose (LoBAG) diets. The general concept is that glucose present in foods is largely responsible for the elevated blood glucose in people with type 2 diabetes. The LoBAG diets are higher in protein and lower in carbohydrate than those recommended by government and professional organizations for the general population. A subscript denotes the percent carbohydrate. Thus, a 30% carbohydrate diet is designated as LoBAG_30_. In all of these diets the protein content is 30% of food energy. When ingested over a 5-week period, a LoBAG_20_ and LoBAG_30_ diet dramatically lowered the 24-hour fasting and 24-hour integrated plasma glucose and glycated hemoglobin, without weight loss, in people with type 2 diabetes [1-3]

In order to further evaluate the metabolic effects of these diets we determined a number of metabolites and hormones [4-6]. The insulin-like growth factor-I (IGF-I) concentration was increased regardless of the carbohydrate content in variations of the LoBAG diet. Since the protein content remained unchanged in all of these diets (30%), we strongly suspect that the increased IGF-I is due to the increased dietary protein content.

The circulating IGF-I concentration is under control of growth hormone (GH) and circulates largely bound in a ternary complex of IGF-I, IGF-Binding Protein 3 (IGFBP-3) and a protein referred to as the Acid-Labile Subunit (ALS). In addition, free IGF-I may be partially regulated acutely by the concentration of IGF-Binding Protein-1 (IGFBP-1). The IGFBP-1 concentration is negatively regulated by insulin. Therefore, we previously evaluated the effect of a LoBAG_30_ diet on GH, IGF-I, IGFBP-3, IGFBP-1, and ghrelin [3]. Ghrelin was measured since it can bind to the GH receptor. It also may be involved in food intake regulation [7,8].

IGF-I increased ~30%, was stable, and independent of a change in GH or IGFBP-3. IGFBP-1 was inversely related to the insulin concentration [3]. The increase in IGF-I and insulin, and decrease in IGFBP-1 theoretically could have a strong anabolic effect, and result in an increase in fat-free mass (FFM). An increase in branched-chain amino acids (BCAAs) derived from the higher dietary protein also could stimulate protein synthesis [9]. However, this could be offset by elevated cortisol and glucagon concentrations [10-13].

We report here additional data from the study in which we previously reported the GH, IGFBPs, insulin and ghrelin data [3]. Specifically, the effect of ingestion of a LoBAG_30_ diet over a 5-week period on the 24-hour circulating total alpha amino acid nitrogen (AAN) (total amino acids), individual specific amino acids, cortisol, and glucagon concentrations is presented. The 24-hour urinary free cortisol was determined as were a number of other circulating and urinary metabolites. The amount of nitrogen ingested during 24 hours and the average of a 4-day fecal nitrogen excretion, as well as 24-hour urinary nitrogen also were quantified. In addition, body composition data were obtained using 4 different methods.

In the aging United States population with and without diabetes, loss of bone and muscle mass, resulting in increased frailty, falls, hip fractures, etc, is a major problem, and an economic burden for the health care system. We posited that if we can demonstrate a positive protein balance, an increase in fat-free mass without a change in weight, and without deleterious effects, a LoBAG diet could have beneficial effects far beyond control of blood glucose in older subjects with type 2 diabetes, and potentially for others as well.

## Methods

Nine male subjects with type 2 diabetes were recruited and screened, as reported previously [2]. Some subjects had been taking oral hypoglycemic medications before enrollment. These were discontinued for varying periods of time to allow the glycated hemoglobin to stabilize before the study was begun. All subjects signed consent forms and all also obtained approval from their primary care provider before discontinuing their medications. Other medications were continued and remained unchanged during the study. The study was approved by the VA Medical Center and University of Minnesota Committees on Human Subjects.

Eight subjects completed the study. One individual participated in a humanitarian aid project during the washout period. He lost a considerable amount of weight during this time, and thus did not complete the second arm of the study. Patient characteristics have been presented previously [3].

A six-day rotating menu was used which was calculated to consist of 30% carbohydrate (low starch), 30% protein, 40% fat (10% saturated fat) (LoBAG_30_) or a control diet (55% CHO, 15% protein, 30% fat) [2,3]. The diets contain foods typical of those consumed by our patients. Total food energy was individualized to insure that each subject remained weight stable during the study. Dietary preferences were accommodated whenever possible. All food was provided to the subjects.

### Protocol

A randomized, crossover, 5 week design with a washout period was used. The age of the subjects ranged from 52 to 70 years. The glycated hemoglobin ranged from 7.1 to 11.4%, with a mean of 8.8%. Subjects were instructed to continue their current activity level during the study. To our knowledge, based on subject interviews, these instructions were followed. The protocol has been described previously [3,6]. Briefly, subjects are admitted to the Special Diagnostic and Treatment Unit (SDTU, similar to a Clinical Research Center) at the beginning and end of each 5-week diet arm. The menu for the control diet that was ingested during the 24-hour data collection period was identical for each of three 24-hour time periods (pre and post control and pre-LoBAG). The LoBAG_30_ menu was specific for the day of the 6-day menu rotation at which the subject underwent the 24-hour blood draw. Meals were fed at 0800 (breakfast), 1200 (lunch), and 1800 (dinner) with a snack at 2100 hr. Blood was obtained at 0730, 0745 and 0800, 15 and 30 minutes after each meal, then at 30 minute intervals for the next 2 hours and finally, hourly until the next meal, for a total of 46 time points for AAN, glucagon and cortisol. Samples for individual amino acids were obtained over 18 hours (3 baseline samples at 0730, 0745 and 0800 and then 7 additional samples taken at 0900, 1000, 1200, 1400, 1800, 2100, 0200 hr). Urine (24 hr) was collected for determination of free cortisol, microalbumin, calcium, creatinine, glucose, pH, potassium, sodium, urea and uric acid. Four day complete fecal specimens were collected with the last day being the day of the 24-hr blood draw.

Although the control diet and the LoBAG_30_ diet were generally isocaloric for each individual subject, the LoBAG_30_ meals were perceived to contain more (“too much”) food. Both diets were readily accepted by the subjects.

### Analytical methods

Plasma and/or urine creatinine, urea nitrogen, sodium, potassium, glucose, uric acid, total cholesterol, HDL-cholesterol, triacylglycerol, pre-albumin and albumin, were determined by an automated method on an Ortho-Clinical Diagnostics Vitros 950 analyzer (Raritan, NJ); LDL cholesterol was calculated with the Friedwald formula; microalbumin was determined using a Beckman-Coulter Array 360 analyzer; urinary calcium and magnesium were determined colorimetrically on a J & J Vitros Instrument (J & J Engineering, Poulsbo, WA). Glucagon was determined by radioimmunoassay using kits purchased from Linco Research (purchased by Millipore, Billerica, MA). AAN, a measure of the total amino acids, was determined by an O-phthaldialdehyde dye binding method (Gusmer Enterprise, Inc Waupaca, WI). Individual amino acid concentrations were determined by high-performance liquid chromatography (ion exchange) using precolumn online derivatization with O-phthalaldehyde and 3-mercaptopropionic acid and 9-fluorenylmethylchloroformate followed by ultraviolet detection. Serum cortisol was determined by Abbott AxSym, based on a Fluorescence Polarization Immunoassay (Abbott Park, IL.). Urinary free cortisol initially was determined in the laboratory of Dr. B. Pearson-Murphy using a high-performance liquid chromatography purification step followed by a cortisol-binding assay (n = 4). Subsequently it has been done by liquid chromatograph, tandem mass spectrometry (LC MS/MS) at Quest, (San Juan Capistrano, CA) (n = 3). It was not determined in one subject.

To determine protein balance, the macronutrient, including protein, content of the meals was calculated using Nutritionist Pro version 2.0 (First data bank: Hearst Corporation, San Bruno, CA). The amount of nitrogen present in the protein was calculated using 5.53 g nitrogen/g protein [14]. The total amount of protein deaminated was determined by quantifying the nitrogen in urine urea nitrogen, creatinine and uric acid excreted over the 24-hrs of the study, the measured nitrogen lost in the feces, and the amount of urea nitrogen retained endogenously. The latter was calculated by determining the difference in plasma urea nitrogen concentration between the fasting baseline at the beginning and at the end of each 24-hour study period, and correcting for plasma water by dividing by 0.94. In this calculation it is assumed that there is a relatively rapid and complete equilibration of urea in total body water. Total body water as a percentage of body weight was calculated using the equation of Watson et al. [15]. The overall assumption is that a change in plasma urea concentration is indicative of a corresponding change in total body water urea concentration. The above calculations did not account for urinary ammonia, amino acids or hippuric acid, which have been reported to result in 1.5 g nitrogen excreted/100 g protein ingested [14]. The grams of nitrogen metabolized have been increased accordingly.

To determine nitrogen balance directly, the nitrogen content of the meals was quantified. Meals for each of the 6 days of the LoBAG_30_ diet, and the one-day of the Standard diet were prepared based on a 2700 Kcal diet and homogenized individually in a Heavy Duty Commercial Warring Blender. Two aliquots for each day were analyzed for nitrogen by thermal conductivity after combustion in an oxygen-rich atmosphere at 850°C. (University of Minnesota Analytical Research Laboratory). Total urinary nitrogen and fecal nitrogen were determined by the Dumas combustion method in the Mayo Clinic Laboratories (Rochester, MN). The amount of urea nitrogen retained in the body water was calculated by determining the difference in plasma urea nitrogen concentration between the fasting baseline at the beginning and at the end of each 24-hour study period, and correcting for plasma water by dividing by 0.94, as above. Total body water was measured directly [16].

Fat-free mass was determined using 3 different techniques, bioelectric impedance, (RJL Systems, Clinton Township, MI), tritiated water dilution [16] (^3^H_2_O from Perkin Elmer, Boston, MA) and Dual-Energy X-Ray Absorptiometry (DEXA), Luna Prodigy DEXA scanner (GE Healthcare, Waukesha, WI). In addition, computed tomography (CT) scans were taken of the abdomen at the level of the L2-L3 interspace and both thighs midway between the iliac crest and the superior margin of the patella in a sitting position at baseline and week 5. Originally a 7.5 mm CT slice was taken. Subsequently the CT scanner was changed and slice thickness was reduced to 6 mm. Data were sent to Dr. Michael Jensen’s laboratory at the Mayo Medical School and Clinic for analysis. Subjects were identified by number only, thus this analysis was done blinded.

For quantification of 24-hour plasma/serum data, net 24-hour incremental area responses were calculated using the overnight fasting value as baseline. Total 24-hour area responses were calculated using zero as the baseline. Both area calculations were done using a computer program based on the trapezoid rule [17]. Statistics were determined by Student's t test for paired variates, comparing the results following 5 weeks on a control diet with those following 5 weeks on a LoBAG_30_ diet. Data are presented as means ± SEM. Prism 4 software was used (Graphpad Software, Inc. San Diego, CA) for the iMac computer (Apple, Cupertino, CA).

## Results

**Figure 1:**
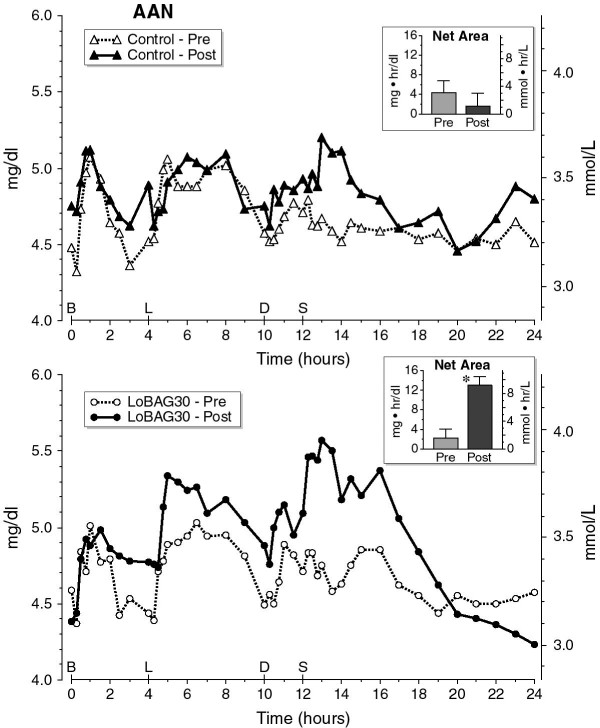
**24 Hour Serum Alpha Amino Nitrogen (AAN) Response (Top – control diet) (Bottom – LoBAG**_**30**_**diet).** The open triangles, broken line represents the mean AAN response at the beginning of the study, while ingesting a control diet of 55% carbohydrate, 15% protein, 30% fat. The closed triangles, solid line represents the AAN response after 5 weeks of ingesting a control diet. The open circles, broken line represents the AAN response at the beginning of the test arm of the study while ingesting a control diet of 55% carbohydrate, 15% protein, 30% fat. The closed circles, solid line represents the AAN response after 5 weeks of ingesting a LoBAG_30_ diet consisting of 30% carbohydrate, 30% protein, 40% fat. The inserts indicate the mean net 24-hour integrated AAN area response pre- and post 5 weeks after ingesting a control (top) or LoBAG_30_ diet (bottom), using the fasting value as a baseline. * indicates statistical significance (P = 0.006).

Fasting AAN concentrations were similar before and after 5 weeks on a control diet, and before and after 5 weeks on a LoBAG_30_ diet (Figure 1). Integrated net area responses were similar before and after 5 weeks on the control diet, but were significantly increased following 5 weeks on a LoBAG_30_ diet, as expected (P < 0.006) (Figure 1 inserts). Integrated total area responses, using zero as baseline, were not statistically different before (115 ± 3 mg.hr/dl) and after 5 weeks (124 ± 2 mg.hr/dl) on a control diet or before (122 ± 5 mg.hr/dl) and after 5 weeks on a LoBAG_30_ diet (124 ± 3 mg.hr/dl) (data not shown).

Integrated 18-hour individual amino acid data before and after being on the control diet and before and after being on the LoBAG diet were determined. In some cases the amino acid concentration was still elevated at 18 hours. The concentration at the 18-hour time point was extrapolated to the fasting baseline to represent the 24-hour time point. The foods ingested during the first 24-hour period of the LoBAG arm were the same as those ingested at the beginning and end of the control arm, i.e. on 3 of the 4 days during which blood was drawn over a 24-hour period, the same foods were provided. Thus, these data have been combined with the control diet data. That is, the control diet data obtained on the 3 occasions have been averaged and compared with the post LoBAG diet data. Nineteen amino acids were quantified. Data for 12 amino acids are presented in Table 1. Data for the other amino acids (aspartate, cysteine, glutamate, histidine, methionine, threonine, tryptophan) are not listed because they are quantitatively minor. In addition, their concentrations did not change. There was considerable variation in all of the results. A statistically significant increase was observed for phenylalanine, tyrosine and the BCAAs. The increase for each BCAA was 5 fold or greater. This also was true for phenylalanine. Leucine increased the most (12 fold).

**Table 1:** **Individual amino acid area response using the average of the three control days μMol**^**.**^**hr/L**

	**Measured 18 hour**		**Extrapolated 24 hour**	
	**Mean of Controls**	**LoBAG**_**30**_	**Mean of Controls**	**LoBAG**_**30**_
Alanine	1203 ± 283	1645 ± 330	1284 ± 297	1742 ± 348
Arginine	23 ± 106	212 ± 165	8 ± 116	200 ± 204
Asparagine	89 ± 31	212 ± 70	100 ± 32	258 ± 86
Glycine	34 ± 90	45 ± 109	38 ± 93	121 ± 115
Isoleucine	115 ± 66	568 ± 63*	133 ± 74	679 ± 82*
Leucine	79 ± 103	966 ± 119*	85 ± 111	1144 ± 149*
Lysine	172 ± 125	548 ± 121	188 ± 147	596 ± 122
Phenylalanine	38 ± 37	225 ± 55*	26 ± 42	259 ± 67*
Proline	739 ± 101	880 ± 107	800 ± 113	969 ± 137
Serine	2 ± 43	28 ± 79	-30 ± 53	40 ± 99
Tyrosine	617 ± 268	1227 ± 266*	652 ± 301	1454 ± 342*
Valine	163 ± 143	1414 ± 175*	168 ± 161	1705 ± 220*

Values are mean ± SEM. * indicates statistically significantly different from the respective control (P < 0.05) based on Student’s t test. Samples were obtained over an 18-hour time period. In some cases the concentration was still elevated at 18 hrs. The concentration from the 18-hour time point was extrapolated to the fasting baseline to represent the 24-hour time point. Those data are presented as “extrapolated” 24-hour data.

**Figure 2:**
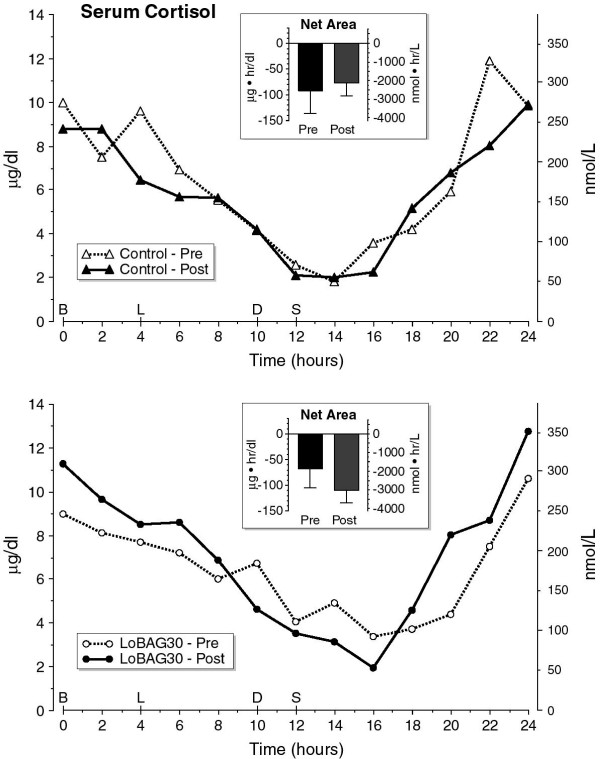
**24 Hour Serum Cortisol Response (Top – control diet) (Bottom – LoBAG**_**30**_**diet).** The open triangles, broken line represents the mean cortisol response at the beginning of the study, while ingesting a control diet of 55% carbohydrate, 15% protein, 30% fat. The closed triangles, solid line represents the cortisol response after 5 weeks of ingesting a control diet. The open circles, broken line represents the cortisol response at the beginning of the test arm of the study while ingesting a control diet of 55% carbohydrate, 15% protein, 30% fat. The closed circles, solid line represents the cortisol response after 5 weeks of ingesting a LoBAG_30_ diet consisting of 30% carbohydrate, 30% protein, 40% fat. The inserts indicate the mean net 24-hour integrated cortisol area response pre- and post 5 weeks after ingesting a control (top) or LoBAG_30_ diet (bottom), using the fasting value as a baseline.

The fasting serum cortisol concentrations were similar before and after 5 weeks on the control diet and before and after 5 weeks on the LoBAG_30_ diet (Figure 2). The post LoBAG (11.3 μg/dl) is not significantly higher (P = 0.184)*.* The concentration decreased from 0800 to a nadir at 2200–2400 hr. It subsequently increased to reach the 0800 hr value again (Figure 2). Neither the integrated net (Figure 2 inserts) nor total area (data not shown) responses were statistically different.

**Figure 3:**
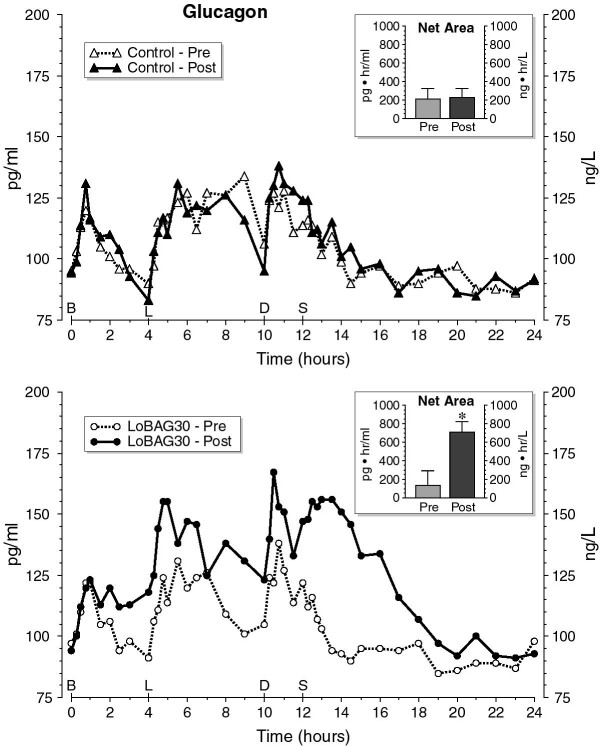
**24 Hour Plasma Glucagon Response (Top – control diet) (Bottom – LoBAG**_**30**_**diet).** The open triangles, broken line represents the mean glucagon response at the beginning of the study, while ingesting a control diet of 55% carbohydrate, 15% protein, 30% fat. The closed triangles, solid line represents the glucagon response after 5 weeks of ingesting a control diet. The open circles, broken line represents the glucagon response at the beginning of the test arm of the study while ingesting a control diet of 55% carbohydrate, 15% protein, 30% fat. The closed circles, solid line represents the glucagon response after 5 weeks of ingesting a LoBAG_30_ diet consisting of 30% carbohydrate, 30% protein, 40% fat. The inserts indicate the mean net 24-hour integrated glucagon area response pre- and post 5 weeks after ingesting a control (top) or LoBAG_30_ diet (bottom), using the fasting value as a baseline. * indicates statistical significance (P = 0.03).

The fasting plasma glucagon concentrations were similar before and after 5 weeks on a control, or on a LoBAG_30_ diet (Figure 3). The integrated net (Figure 3 insert) (210 ± 111, 226 ± 96 and 133 ± 158 pg hr/ml) and total area responses (2501 ± 276, 2309 ± 303, 2455 ± 235 pg hr/ml) were similar before and after the control diet and before the LoBAG_30_ diet, respectively. However, both the integrated net (708 ± 115 pg hr/ml) and total (2958 ± 249 pg hr/ml) (not shown) glucagon area responses were significantly increased while on the LoBAG_30_ diet (p = 0.03 and p = 0.01, net and total area responses, respectively).

Blood pressure, serum creatinine, uric acid, albumin, pre-albumin and HDL cholesterol were unchanged after 5 weeks on either the control or the LoBAG_30_ diet (Table 2). Total and LDL cholesterol were significantly decreased after 5 weeks on the LoBAG_30_ diet. Triglycerides were significantly decreased after 5 weeks on either diet..

**Table 2:** Caloric Intake, Blood Pressure, Plasma/Serum Metabolites

	**Control-Pre**	**Control-Post**	**LoBAG-Pre**	**LoBAG-Post**
Caloric Intake	2513 ± 135	2613 ± 134	2619 ± 106	2612 ± 144
Blood Pressure				
Systolic	140 ± 9	134 ± 9	139 ± 4	131 ± 6
Diastolic	83 ± 4	78 ± 3	78 ± 3	78 ± 4
Creatinine (mg/dl)	1.0 ± 0.05	1.0 ± 0.06	1.0 ± 0.05	1.0 ± 0.07
Cholesterol (mg/dl)	156 ± 14	134 ± 8	166 ± 12	140 ± 12 *
HDL (mg/dl)	39 ± 2	35 ± 2	36 ± 2	35 ± 2
LDL (mg/dl)	92 ± 10	80 ± 6	102 ± 12	86 ± 10 *
Triglycerides (mg/dl)	142 ± 24	94 ± 12 *	138 ± 19	97 ± 17 *
Serum Uric Acid (mg/dl)	5.2 ± 0.4	4.7 ± 0.3	5.1 ± 0.3	5.0 ± 0.4
Albumin (mg/dl)	4.5 ± 0.05	4.4 ± 0.08	4.4 ± 0.07	4.4 ± 0.07
Pre albumin	25.0 ± 1.3	24.3 ± 1.1	25.3 ± 1.3	24.7 ± 1.0

Both Control and LoBAG diets were designed to contain ~10% saturated fatty acids.

Values are Means ± SEM.

Statistics were done using Student’s t test comparing Control-Pre with Control-Post and LoBAG-Pre with LoBAG-Post. * p ≤ 0.05

Urinary excretion of sodium, urea nitrogen, uric acid, creatinine, and the creatinine clearance, as well as the urinary free cortisol all were significantly increased following 5 weeks on a LoBAG_30_ diet compared to the pre-5 week baseline. Urine volume, potassium and calcium were similar before and after either the control or the LoBAG_30_ diet (Table 3).

**Table 3:** Urine Data (n = 8)

	**Control- Pre**	**Control -Post**	**LoBAG-Pre**	**LoBAG-Post**
**Volume (ml)**	4171 ± 654	3889 ± 875	4671 ± 655	4500 ± 581
**Glucose (g)**	10 ± 6	7 ± 3	16 ± 8	3 ± 2 **~**
**Potassium (m atoms)**	91 ± 10.8	78 ± 7.6	103 ± 11.8	98 ± 5.6
**Sodium (m atoms)**	188 ± 15	211 ± 20	201 ± 15	273 ± 15 *
**Urea Nitrogen (g)**	13.6 ± 1.1	13.4 ± 0.9	15.8 ± 1.0	22.5 ± 0.7 *
**Uric Acid (g)**	0.74 ± 0.04	0.69 ± 0.03	0.74 ± 0.05	0.90 ± 0.03 *
**Urine Nitrogen ** **(Urea, Creat, Uric Acid only)**	14.5 ± 1.1	14.3 ± 0.9	16.7 ± 1.0	23.6 ± 0.7*
**Micro albumin (mg)**	< 5	< 5.0	< 5.0	< 5.0
**Cortisol (μg) ( n = 6)**	17.9 ± 4.8	21.6 ± 6.4	24.6 ± 4.6	42.8 ± 3.3*
**Calcium (g)**	263 ± 30	251 ± 34	265 ± 37	262 ± 42
**Creatinine (g)**	1.8 ± 0.09	1.7 ± 0.06	1.9 ± 0.11	2.2 ± 0.15*
**Creatinine Cl (ml/min)**	128 ± 10	127 ± 10	131 ± 8	160 ± 12*
**pH**	5.9 ± 0.2	6.3 ± 0.2*	6.2 ± 0.2	6.1 ± 0.1

Values are mean ± SEM.

Statistics were done using Student’s t test comparing Control – Pre with Control – Post, and LoBAG – Pre with LoBAG – Post.

* = P < 0.05 using Student’s t test compared to Pre;

~ P = 0.06 using Student’s t test, and P = 0.008 using Wilcoxon’s sign rank test.

The 24-hour fecal nitrogen excreted before and after 5 weeks on a control diet and before 5 weeks on a LoBAG_30_ diet was similar (1.6 ± 0.2, 1.7 ± 0.2, and 1.7 ± 0.3 g/day, respectively). It was significantly greater after 5 weeks on a LoBAG_30_ diet (2.2 ± 0.3 g/day p = 0.04).

### Calculated protein balance

The protein balance (Table 4, A) during the 24-hour study periods at the end of each 5-week period was calculated as we have done before [5,6] with the following exceptions. Previously, only the nitrogen in urine urea was accounted for. In the present study the nitrogen contribution of urine creatinine and uric acid also were included. Also, fecal nitrogen was determined directly, and a correction was made for excreted ammonia, amino acids, and hippuric acid (1.5 g) [14].

**Table 4:** Protein (Nitrogen) Balance

	**Ingested**		**Metabolized**		**% Metab**
	g Nitrogen	g Protein	g Nitrogen	g Protein	
**A**	Calculated	Computer Tables	Measured	Calculated	
Control Pre	17.0	94.2 ± 5.1	16.0 ± 1.0	88.5	94
Control Post	17.7	98.0 ± 5.0	16.9 ± 1.1	93.5	96
LoBAG Pre	17.7	98.2 ± 4.0	18.1 ± 1.3	100.1	102
LoBAG Post	35.4	195.9 ± 10.8	26.5 ± 11	146.5	75
LoBAG Post*	31.3	195.9 ± 10.8	26.5 ± 11	165.6	85
	**Ingested**		**Metabolized**		**% Metab**
	g Nitrogen	g Protein	g Nitrogen	g Protein	
**B**	Measured	Calculated	Measured	Calculated	
Control Pre	17.4 ± 0.9	96.2	20.1 ± 1.3	112.2	116
Control Post	18.1 ± 0.9	100.1	21.5 ± 2.0	118.9	119
LoBAG Pre	18.1 ± 0.7	100.1	20.3 ± 1.0	112.3	112
LoBAG Post	31.8 ± 2.5	175.9	29.4 ± 1.5	162.6	92

A. Protein content of the meals was estimated using Nutritionist Pro version 2.0: (First data bank: Hearst Corporation, San Bruno, CA); Nitrogen in food was calculated using 5.53 g protein/g N as a conversion factor [14]; *Nitrogen in food in the LoBAG post diet also was calculated using the conventional 6.25 g protein/g N as a conversion factor, since the majority of the additional protein in the diet was animal protein (muscle); body water was calculated according to Watson [15]; total urine nitrogen was determine by summing the urine urea nitrogen concentration,% nitrogen in creatinine (37%) and% nitrogen in uric acid (33%). Fecal nitrogen was measured directly. The contribution of nitrogen from excreted ammonia, amino acids and hippuric acid was added as indicated in methods.

B. Protein content of the meals was determined directly; body water was determined directly; total urine nitrogen and fecal nitrogen were determined directly (see text for methods).

% Metabolized was calculated as g N (Metabolized/g N Ingested) × (100).

On the first and the last day of the 5 weeks on the control diet, the calculated protein ingested based on published data (Nutritionist Pro, version 2.0) was 94.2 ± 5.1 g and 98.0 ± 5.0 g, respectively. The metabolized protein was accounted for by calculation of **1)** retention of urea nitrogen in body water, **2)** the nitrogen measured in urine urea, creatinine and uric acid, and **3)** the nitrogen contribution from ammonia, amino acids, and hippuric acid [14]. The calculated values at the beginning and end of the control diet were 88.5 ± 6.7 g and 93.5 ± 5.4 g, (94 and 96%) respectively. When ingesting the control diet on the first day of the LoBAG_30_ arm of the study, 98.2 ± 4.0 g of protein was calculated to have been ingested and 100.1 ± 6.4 g was accounted for by retained urea nitrogen and that excreted in urine and feces (102%). After 5 weeks on the LoBAG_30_ diet, the calculated protein ingested was 195.9 ± 10.8 g; 146.5 ± 4.3 g were accounted for by metabolism (75%). Thus, the calculated protein actually ingested was similar for all 3 control diets, as expected. The nitrogen balance was essentially neutral on these control diets (mean = 97%). In contrast, when the LoBAG_30_ diet was ingested, the protein balance clearly was positive (Table 4, A) (P = 0.01).

#### Measured nitrogen (protein) balance

When the actual nitrogen content of the food was determined by a thermal conductivity method, the mean total nitrogen content in the control diets was similar to that calculated (Table 4 B vs A). Although there are numerous nitrogen containing compounds in food, other than protein [18], these are present in relatively small amounts in food. [19-21]. Interestingly, the determined nitrogen content of the LoBAG_30_ diet was lower than the value calculated based on protein intake (31.8 g vs 35.4 g, respectively, Table 4 B vs A).

During the review process of the manuscript, one Reviewer pointed out that if the factor 6.25 is used in the conversion of nitrogen to protein, the calculated protein intake is 31.3 g, which makes the value very similar to the 31.8 g measured. The increase in dietary protein in the LoBAG diet *was* largely due to an increase in meat protein. The traditional calculated nitrogen in meat is 16% (6.25), rather than 19% (5.53). These factors (6.25 and 5.53) were used to calculate the protein, based on nitrogen measured.

Overall, the actually determined nitrogen excreted was considerably higher than the actually measured nitrogen in the food ingested for the Control-Pre, Control-post and LoBAG-Pre data sets (Table 4). The reason for this is unexplained. However, it should be noted that a different method was used in the determination of the fecal and urine nitrogen compared to food nitrogen (Table 4).

The determined nitrogen data (Table 4B) resulted in a markedly negative protein balance when the subjects ingested the control diet, as reported previously by others [22]. Nevertheless, both for the calculated as well as the measured nitrogen balance, the balance was positive for the LoBAG_30_ diet. Indeed the difference from the average percent of protein metabolized of the three control diets (Control pre, Control – post, LoBAG – pre) and the LoBAG post, was nearly identical, whether protein balance was determined by method A (Mean 97 – 75 = ∆22%) or method B (Mean 116 – 92 = ∆ 24%)

Overall the data clearly indicate a relative difference in nitrogen balance when the subjects ingest a LoBAG_30_ diet compared to the control diet.

### Body composition

Body composition data are presented in Table 5. Subjects total body weights were similar before or after ingesting either diet. The fat-free mass (FFM) obtained by DEXA correlated well with the tritium dilution data, but both were lower than with Bioelectric Impedance. The mean FFM was similar before and after ingesting the control or the LoBAG diet.

**Table 5:** Body Composition

	**Control–Pre**	**Control–Post**	**LoBAG**_**30**_**Pre**	**LoBAG**_**30**_**Post**
Total Body Weight (lbs)	215 ± 5.7	214 ± 6.3	215 ± 5.6	215 ± 6.3
Kg	97.6	97.2	97.2	97.2
	**Kg Fat-Free Mass (n = 8)**			
DEXA	63 ± 2	62 ± 2	64 ± 2	64 ± 2
^3^H_2_O	63 ± 2	63 ± 3	67 ± 2	68 ± 2
Bioelectric Impedance	74 ± 2	72 ± 2	74 ± 2	74 ± 2
Creatinine Excretion **	61 ± 3	58 ± 2	61 ± 3	73 ± 4*
	**CT Scan (cm**^**2**^**) (n = 7)**			
Visceral Abdominal Fat	275 ± 23	249 ± 23	264 ± 22	247 ± 25
Total Abdominal Fat	504 ± 41	460 ± 40	492 ± 42	456 ± 45
Right Thigh Fat	71 ± 10	67 ± 9	71 ± 11	75 ± 12
Left Thigh Fat	70 ± 10	64 ± 7	68 ± 10	71 ± 11
Right Thigh Muscle	167 ± 8	165 ± 8	168 ± 9	168 ± 9
Left Thigh Muscle	162 ± 8	162 ± 8	164 ± 9	164 ± 10

Values are Mean ± SEM. * P = 0.0009.

** Method of Forbes and Bruining [23].

When using creatinine excretion as an indirect measurement of FFM (particularly muscle mass) [23], it was significantly increased. The increase could not be explained by the creatine/creatinine or uric acid derived from the additional meat and other protein sources in the diet [24]. It also could not be attributed to the increase in creatinine clearance since the mean plasma creatinine was unchanged. Also, creatinine, like urea, rapidly diffuses throughout body water and is not an issue.

The CT data also indicated no change in visceral, total abdominal or thigh fat and no change in thigh muscle area.

## Discussion

As indicated previously, a LoBAG diet theoretically could result in a positive protein balance and an increase in FFM for several reasons. First, the postprandial insulin response to the LoBAG_30_ diet is vigorous [3] and insulin is reported to decrease whole body protein degradation and possibly stimulate protein synthesis [9,25,26], although this effect is blunted in men with type 2 diabetes [27].

Second, total IGF-I is increased by ~ 30% and free IGF-I is likely to be increased further because of a marked decrease in IGFBP-1 associated with meal ingestion [3]. That the total IGF-I was increased but IGFBP-3 was not [3] also suggests that the additional IGF-I was either bound to other IGF-I binding proteins [3] and/or the free IGF-I was markedly elevated when the subjects were ingesting the LoBAG diet.

The effect of IGF-I on protein anabolism has been reported to occur only at high and perhaps unphysiological concentrations, and was attributed to its insulinmimetic activity [28]. However, others have clearly reported that IGF-I stimulates skeletal muscle protein synthesis at physiological levels, an effect independent of its insulin-like effect [9].

In addition, IGF-I is reported to not only stimulate protein synthesis and to accelerate amino acid clearance [9,29-32] but possibly to inhibit degradation as well [33]. Whether the in vivo metabolic effects of IGF-I are independent of or depend on it being bound to carrier proteins remains an unresolved issue [34].

Third, an increase in circulating total amino acid concentration and specifically BCAAs stimulate protein synthesis [9,27]. A maximal stimulation was reported to occur with an increase in total amino acids of 2–3 fold [26]. In the present study, the 24 hour integrated net area in the 3 controls was increased 2-4 fold, and ~13 fold with a LoBAG_30_ diet, (>4 times that in the controls)**.** There also was a large increase in BCAAs, particularly leucine (12 fold) which, in the presence of a high insulin concentration, should strongly stimulate net protein accumulation [25,26,35], and it was sustained for a prolonged period of time.

In contrast, an increased cortisol and glucagon concentration could oppose the anabolic stimulation by insulin, IGF-I, total and BCAAs. The 24-hour cortisol profile remained unchanged (Figure 2). However, the 24-hour urinary free cortisol was increased (Table 3). The latter suggests that the free, presumably active form of circulating cortisol, could have been increased. We previously observed an increase in urinary free cortisol in subjects ingesting a LoBAG_20_ diet but this was not statistically significant. We did not do a 24-hr serum cortisol profile in that study [6].

We were somewhat surprised that the serum total cortisol was not increased because several years ago we demonstrated a post-meal increase in cortisol and adrenocorticotropin (ACTH) when young, normal subjects ingested a diet containing 4 g protein/kg body weight over a 12-hour period in the form of 3 identical meals [36]. A small but significant increase also occurred when the protein content was 1 g/kg body weight. That an increase did not occur in the present study suggests that the protein content was not sufficient for an increase to be observed, or more likely, the subjects being older, more obese and with type 2 diabetes, do not respond to an increase in dietary protein as well as do young people without diabetes. It also is possible that a metabolic adaptation to the increased protein content occurred over 5 weeks. The previous study was only a single day study. Nevertheless, a subtle increase may have been present in the current study as indicated by the increase in urinary free cortisol.

That the fasting glucagon concentration was unchanged in the present study was expected. That the 24 hr integrated net and total glucagon area responses were further elevated (5 fold and 20%, respectively) as a consequence of ingestion of the LoBAG_30_ diet was somewhat unexpected (Figure 3). Ingestion of a LoBAG_30_ diet in a previous study resulted in only a modest and not significant further elevation in glucagon when compared to the control diet (15% protein) [2]. However, a clearly elevated net area response (2.6 fold) was present when the carbohydrate content was reduced to 20% in a LoBAG_20_ diet [1]. We previously reported that high carbohydrate diets modestly decrease the circulating glucagon, in normal young subjects, whereas high protein or high fat diets greatly increased it in single day studies. However, with the typical ratios found in the American diet (45–55% CHO, 10-15% Pro) there generally is little change in glucagon concentrations after meals [8,37].

An elevated glucagon concentration has been reported to increase amino acid clearance and stimulate urea synthesis, whereas a raised glucose concentration inhibits it [38]. Thus, the metabolic disposal of the increased absorbed amino acids derived from ingesting the LoBAG diet could have been accelerated by the increased glucagon concentration. The total amino acid concentration itself also has been reported to regulate the urea synthesis rate in a concentration-dependent fashion (reviewed in [38]).

Glucagon given as an IV bolus, also has been reported to stimulate a transient rise in IGFBP-1 [39]. However, even though the 24 hour net glucagon concentration increased 5 fold in the present study (Figure 3) the IGFBP-1 decrease was similar to that when the subjects ingested the control diet [3].

As we have reported previously, a LoBAG_30_ diet strongly stimulates an increase in insulin [1-3]. The insulin concentration is further increased relative to the glucose concentration. Thus, the calculated insulin resistance also is increased. We consider this resistance to be physiologic and to be due to the increased protein as well as fat content. In any regard, the increase in insulin stimulated by protein ingestion but without a decrease in glucose into the hypoglycemic range is important since it allows the insulin to inhibit proteolysis, and thus facilitate a net increase in protein synthesis stimulated by the increased BCAAs, particularly leucine. In addition, a modest increase in cortisol could induce a mild insulin-resistant state and thus, limit the effect of insulin on glucose metabolism. The ingested protein-stimulated increase in glucagon facilitates deamination of amino acids and urea formation i.e. it facilitates the disposition of the remaining amino acids [38]. The net effect is to rapidly remove from the circulation those absorbed amino acids not removed by the gut cells. The deaminated amino acids are largely converted to glucose through gluconeogenesis and could replace endogenous gluconeogenic substrates [40,41]. The result of the entire integrated process is the amino acids are disposed of relatively rapidly without a significant change in plasma glucose [42-44]. Overall, it facilitates body protein homeostasis or in the case of a LoBAG diet, a positive nitrogen balance and an eventual possible modest increase in protein mass.

Of considerable interest, even though the increase in total serum amino acid net area response was approximately 4 fold greater when the subjects ingested a LoBAG_30_ diet (Figure 2), the total amino acid concentration had returned to the fasting baseline by the following morning indicating complete disposal, either through metabolism (deamination) and/or from incorporation into protein.

Amino acids also can be temporarily stored in skeletal muscle [45], but this is not likely to have been of importance. It also has been reported that during the day not only are the diet-derived amino acids oxidized but additional new protein is synthesized due presumably to the increase in insulin, BCAAs, etc. and to which an increase in free IGF-I, as indicated here, could contribute. During the night net proteolysis results in a stable protein mass [46]. This may be the case even when the protein content is increased as in the current study.

Data obtained in this and previous studies indicated that the higher content of protein (30%) in a LoBAG diet resulted in a relatively large calculated positive protein balance, whereas the control diet, (55% carbohydrate, 15% protein, 30% fat), resulted in a calculated neutral balance [6]. However, in the present study, the dietary nitrogen was directly quantified, and not just calculated from food tables. In addition to the urinary nitrogen used in our calculation of protein balance, the fecal nitrogen also was quantified. These directly measured results indicate that the net protein balance, as estimated from nitrogen balance, was negative with the control diet, but was positive with a LoBAG_30_ diet.

A more accelerated loss of appendicular lean body mass and an increased risk of sarcopenia with aging has been reported in people with type 2 diabetes [47,48]. That is, nitrogen balance is negative and greater than in those without diabetes. However, this change in balance would be subtle and far less than anticipated with the negative nitrogen balance observed here.

The current data do not indicate an overall increase in fat-free mass and presumably protein mass (Table 5). The CT data also did not indicate an increase in muscle mass or a reduction in thigh and abdominal fat. However, the urinary creatinine data suggest an increase in muscle mass may have occurred [23], although not sufficient for it to be detected over this time frame. Thus, a larger and longer duration study is needed to determine if the demonstrated positive nitrogen balance results in a demonstrable increase in muscle mass and function.

Regarding the time duration required to observe a change in lean mass or muscle mass, a decrease in mid thigh muscle was determined by CT in 10 healthy men and women (age 55–77 years) after 14 weeks on a low protein (0.8 g/kg/day) diet [49]. Also in healthy male subjects, Westerterp-Plantera and associates have reported a positive protein balance and a negative fat balance in a 4 day comparison of a 30% vs. 10% protein diet [50]. The same group reported an increase in fat free mass of 0.73 Kg (1.6 pounds) independent of a change in body weight over a 3 month increased dietary protein intervention. It also resulted in a negative fat balance [51]. In another study, an increase in muscle mass was observed within 4 weeks when testosterone was administered during exercise training [52].

## Conclusion

The LoBAG_30_ diet, a high protein, low carbohydrate diet, ingested over 5 weeks results in an in vivo metabolic and hormonal milieu in which a positive protein balance would be expected. The protein balance *was* positive, but an increase in FFM was not observed. It is possible that a subtle increase in lean body metabolizing mass was present. Proof will require a larger and longer-term study. In any regard, at present it is not possible to account for the calculated and determined positive protein balance by an increase in FFM. It is possible that a systematic analytical or calculation error is present. If so, how this occurred is not apparent.

## Competing interests

The authors declare that they have no competing interests.

## Authors’ contributions

The authors contributed equally to the study i.e. experimental design, obtaining funding, analyzing data and writing the manuscript. Both authors read and approved the final manuscript.

## Funding

Supported in part from merit review funds from the Department of Veterans Affairs, and grants from The National Pork Board, the Minnesota Beef Council and the National Cattlemen’s Beef Association, funded by “The Beef Checkoff.”

The study is filed in ClinicalTrials.gov (NCT00108225).
